# Supplementary material for: Application of copper(II)-based chemicals induces CH3Br and CH3Cl emissions from soil and seawater
Source: Nat Commun. 2022 Jan 10;13:47. doi: 10.1038/s41467-021-27779-3 (PMC8748922; doi:10.1038/s41467-021-27779-3)
Supplement: Supplementary file 1 — Supplementary Information [file 41467_2021_27779_MOESM1_ESM.pdf]

Supplementary Information for

Application of copper(II)-based chemicals induces CH_3Br and CH_3Cl emissions from soil and seawater

Yi Jiao[1,4,*], Wanying Zhang[2], Jae Yun Robin Kim[1], Malte Julian Deventer[1,5], Julien Vollering[1,6], and Robert C. Rhew[1,3,*]

1. Department of Geography, University of California, Berkeley, California, 94720, USA

2. Anhui Province Key Laboratory of Polar Environment and Global Change, School of Earth and Space Sciences, University of Science and Technology of China, Hefei, 230026, China

3. Department of Environmental Science, Policy and Management, University of California, Berkeley, California, 94720, USA

4. Present address: Terrestrial Ecology Section, Department of Biology, University of Copenhagen, Universitetsparken 15, Copenhagen Ø, DK-2100, Denmark

5. Present address: ANECO Institut für Umweltschutz GmbH & Co., Hamburg, 21079, Germany

6. Present address: Department of Environmental Sciences, Western Norway University of Applied Sciences, 5020 Bergen, Norway

* Correspondence should be addressed to Y.J. (yi.jiao@bio.ku.dk) or R.C.R (rrhew@berkeley.edu)

Table of content

Five figures –

Supplementary Fig. 1 CH_3Br and CH_3Cl fluxes from experiment IV

Supplementary Fig. 2 Photographs of the chemical mixtures after 2 hours of reaction in experiment IV

Supplementary Fig. 3 CH_3Cl fluxes from experiment V

Supplementary Fig. 4 A representative photograph of the mineral soil and autoclaved soil used in this study

Supplementary Fig. 5 Presumed degradation pathway of catechol by Fenton-like or photo-Fenton reactions based upon this study and literature

Four tables –

Supplementary Table 1 Soil biogeochemical properties at the Oxford Tract

Supplementary Table 2 Temperature, UVA, UVB, and photosynthetic active radiation (PAR) intensities during the light experiment III

Supplementary Table 3 Temperature, UVA, UVB, and photosynthetic active radiation (PAR) intensities during the light experiment IV

Supplementary Table 4 Other possible products detected in experiment IV

One note –

Supplementary Note 1 A simplified calculation on CH_3Br and CH_3Cl emissions with respect to the global agricultural application of copper(II) sulfate

Supplementary References

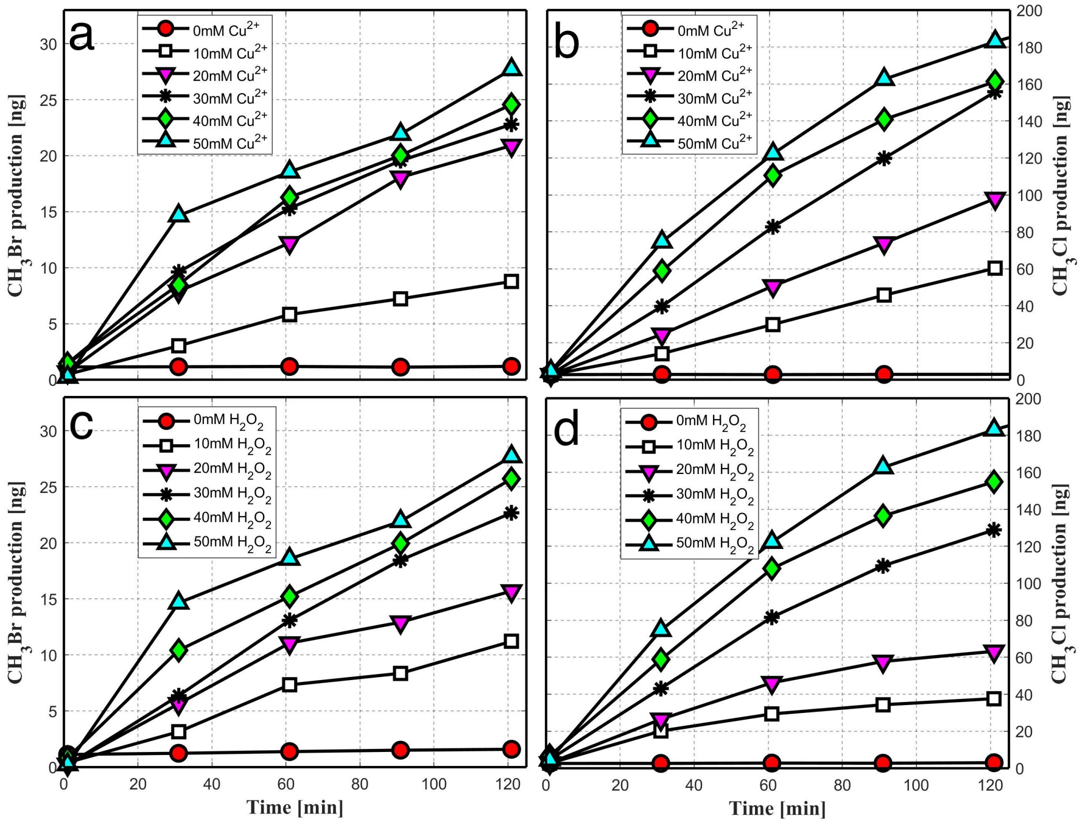

Supplementary Fig. 1 Methyl bromide (CH_3Br) and methyl chloride (CH_3Cl) production over time from different chemical mixtures: (**a**) 10 ml each of 10 mM catechol, 20 mM KBr, 50 mM H_2O_2, and $CuSO_4$ at concentrations of 0 mM, 10 mM, 20 mM, 30 mM, 40 mM and 50 mM; (**b**) 10 ml each of 10 mM catechol, 20 mM KCl, 50 mM H_2O_2, and $CuSO_4$ at concentrations of 0 mM, 10 mM, 20 mM, 30 mM, 40 mM and 50 mM; (**c**) 10 ml each of 10 mM catechol, 20 mM KBr, 50 mM $CuSO_4$, and H_2O_2 at concentrations of 0 mM, 10 mM, 20 mM, 30 mM, 40 mM and 50 mM; (**d**)10 ml each of 10 mM catechol, 20 mM KCl, 50 mM $CuSO_4$, and H_2O_2 at concentrations of 0 mM, 10 mM, 20 mM, 30 mM, 40 mM and 50 mM. The figure showed the average values of two replicative measurements for each treatment (n = 2). The individual fluxes is accessible in "Data availability" in the main text.

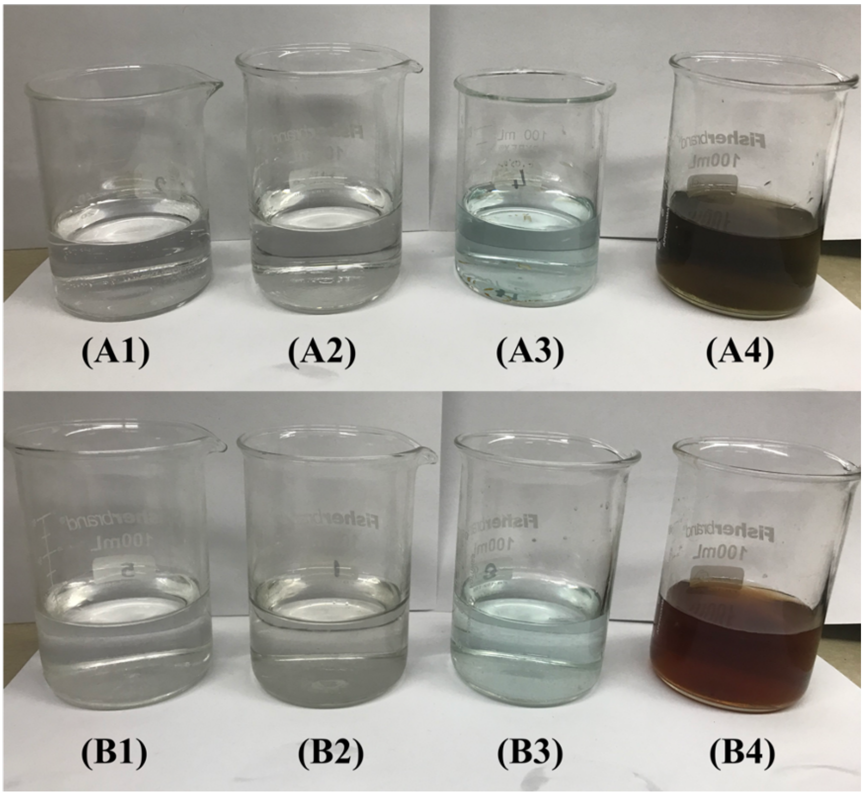

Supplementary Fig. 2 Photographs of the chemical mixtures after ~2 hours of reaction at 20° C. The configurations of the chemical mixtures were: (**A1**) 10 ml 10 mM catechol (benzene-1,2-diol), 10 ml 20 mM KCl, 20 ml H_2O; (**A2**) 10 ml 10 mM catechol, 10 ml 20 mM KCl, 10 ml 50 mM H_2O_2, 10 ml H_2O; (**A3**) 10 ml 10 mM catechol, 10 ml 20 mM KCl, 10 ml 50 mM $CuSO_4$, 10 ml H_2O; (**A4**) 10 ml 10 mM catechol, 10 ml 20 mM KCl, 10 ml 50 mM H_2O_2, 10 ml 50 mM $CuSO_4$; (**B1**) 10 ml 10 mM catechol, 10 ml 20 mM KBr, 20 ml H_2O; (**B2**) 10 ml 10 mM catechol, 10 ml 20 mM KBr, 10 ml 50 mM H_2O_2, 10 ml H_2O; (**B3**) 10 ml 10 mM catechol, 10 ml 20 mM KBr, 10 ml 50 mM $CuSO_4$, 10 ml H_2O; (**B4**) 10 ml 10 mM catechol, 10 ml 20 mM KBr, 10 ml 50 mM H_2O_2, 10 ml 50 mM $CuSO_4$.

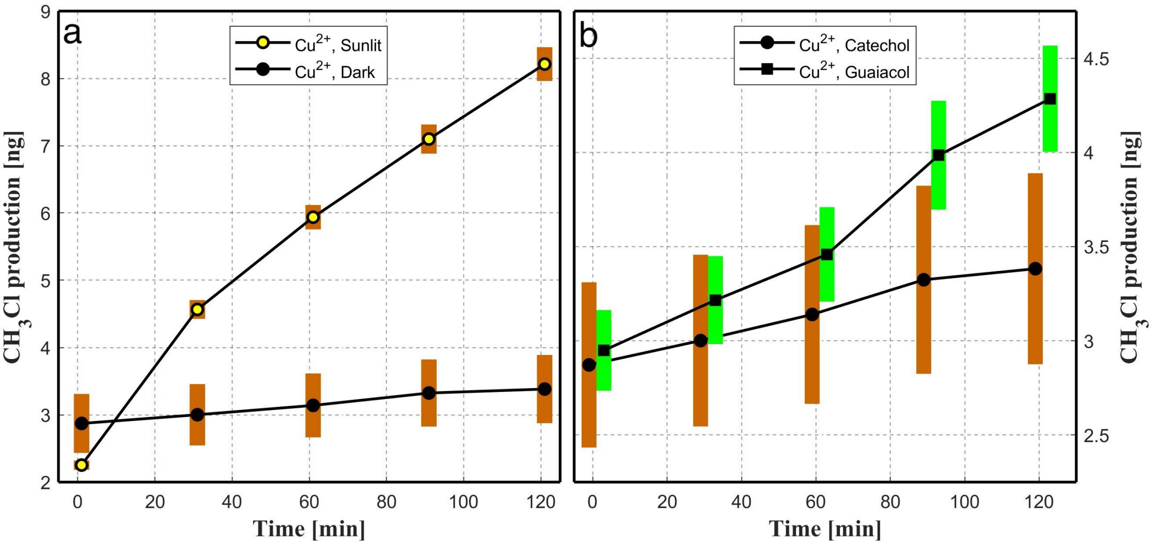

Supplementary Fig. 3 Methyl chloride (CH_3Cl) production over time from different chemical mixtures: (**a**) 10 ml each of 10 mM catechol, 20 mM KCl, and 50 mM CuSO4, mixed and incubated under either sunlit or dark conditions; (**b**) 10 ml each of either 10 mM catechol or 10 mM guaiacol, and 20 mM KCl, 50 mM $CuSO_4$, mixed and incubated under dark conditions. The symbols represent the average values of two replicate incubations (n = 2) and the upper and lower boundary of the bars represent the two individual fluxes.

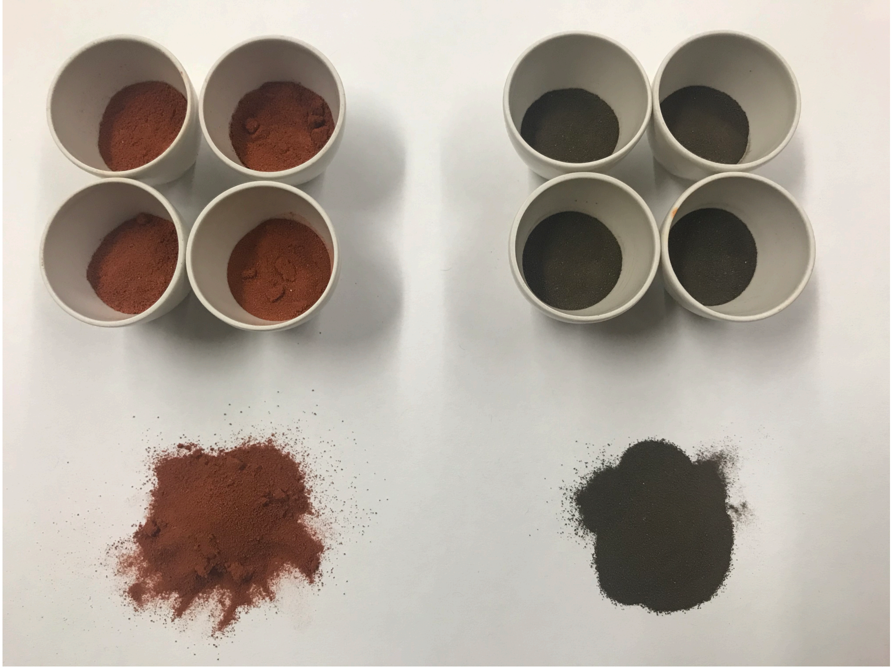

Supplementary Fig. 4 Photograph of a representative set of mineral soil (left) and autoclaved soil (right) samples used in this study. The reddish-brown color of the mineral soil shows the depletion of organic matter after the "Loss on Ignition" combustion process, while the dark color of the autoclaved soils shows the preservation of organic matter.

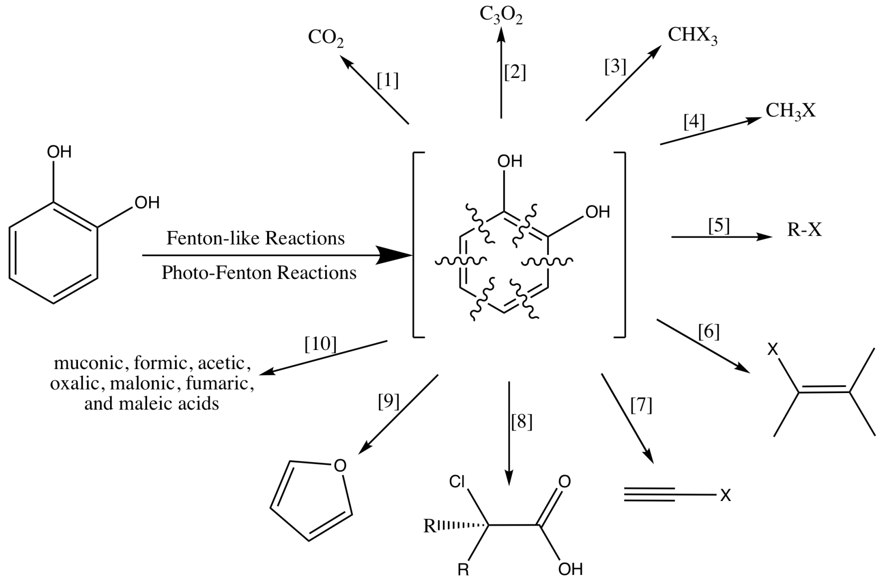

Supplementary Fig. 5 Presumed degradation pathway of catechol by Fenton-like or Photo-Fenton reactions[1] to form [1] carbon dioxide[2], [2] carbon suboxide[3], [3] trihalomethanes[4], [4] methyl halides, [5] chloroethane, idioethane and other chloroalkanes[5–7], [6] chloroethene, [7] chloroethyne[6], [8] chlorinated acetic acid[8,9], [9] furan[10,11], and [10] muconic, formic, acetic, oxalic, malonic, fumaric and maleic acids[2,12–14]. X represents Cl, Br or I. For the presumed pathways catalyzed by Cu(II), future experiment is needed to measure Cu(II) and Cu(I) concentrations to verify their roles during the reaction.

Supplementary Table 1. Soil biogeochemical properties at the Oxford Tract, including organic matter, element contents, pH, and soil texture, etc. Analyses conducted by A&L Western Agricultural Laboratories[a], Modesto, California, U.S. Chloride and bromide data was from another study[15], which used the soil from the same field site as this study.

**Analyte [unit]**	**Results**	**Analyte [unit]**	**Results**
Organic Matter [%]	4.7	Chloride Cl^- [ppm]	148 ± 71
NO_3-N [ppm]	44.5	Bromide Br^- [ppm]	1.1 ± 0.3
Phosphorus Weak Bray [ppm]	149	Iron [ppm]	63
$NaHCO_3$-P [ppm]	74.5	Copper [ppm]	5.4
Potassium K [ppm]	278	CEC [meq/100g]	22.7
Magnesium Mg [ppm]	437	Sand [%]	42
Calcium Ca [ppm]	2531.5	Silt [%]	24
Sodium Na [ppm]	26.5	Clay [%]	34
Sulfur SO_4-S [ppm]	5.5	Texture	Clay Loam
pH	5.6		

[a] About A&L Western Agricultural Laboratories http://www.al-labs-west.com/

Supplementary Table 2. Temperature, UVA (λ = ~320 to 390 nm, half-sensitivity points, resolution 5 mW m^{-2}, Vernier UVA-BTA), UVB (λ = 265-322 nm, 10% sensitivity points, resolution 0.25 W m^{-2}, Vernier UVB-BTA) and **p**hotosynthetic **a**ctive **r**adiation (PAR, λ = 410-655 nm, resolution 1 μmol m^{-2} s^{-1} (1 W m^{-2} ≈ 4.6 μmol m^{-2} s^{-1})[b], absolute accuracy ±5%, Vernier PAR-BTA) intensities inside the glass jar during the light treatment in experiment III (n = 3). Means and standard deviations are rounded based upon the resolution of measurements.

Day	Temperature [°C]	UVA [mW m^{-2}]	UVB [mW m^{-2}]	PAR[μmol m^{-2} s^{-1}]
1	22.7 ± 0.8	19370 ± 420	96 ± 11	870 ± 50
2	19.5 ± 1.1	19540 ± 100	82 ± 14	790 ± 150
3	16.3 ± 1.0	19330 ± 270	38 ± 16	710 ± 40
4	19.7 ± 0.5	19230 ± 110	44 ± 12	780 ± 110
5	21.7 ± 0.7	19500 ± 250	249 ± 38	950 ± 90
6	26.4 ± 1.9	19380 ± 270	457 ± 34	1860 ± 90
7	27.6 ± 0.7	19410 ± 50	506 ± 9	1920 ± 150
8	26.1 ± 0.7	19360 ± 230	490 ± 31	1940 ± 80
9	-	19220 ± 180	600 ± 12	2110 ± 100
10	-	19460 ± 220	256 ± 39	1060 ± 70
11	-	19300 ± 260	439 ± 48	1690 ± 210
12	-	19420 ± 420	61 ± 20	520 ± 90
13	-	19610 ± 260	358 ± 15	1480 ± 210
14	-	19500 ± 360	591 ± 42	1930 ± 70
15	-	19180 ± 170	416 ± 90	1340 ± 240
16	-	19220 ± 280	515 ± 36	1770 ± 120
17	-	19290 ± 210	410 ± 188	1540 ± 510

[b] An approximation assuming a flat spectral distribution curve over 400-700 nm is 1 W m^{-2} ≈ 4.6 μmol m^{-2} s^{-1} (https://www.licor.com/env/pdf/light/Rad_Meas.pdf).

Supplementary Table 3. Temperature, UVA, UVB, and PAR intensities inside and outside the glass jar during the light treatment in experiment V (n = 10). Means and standard deviations are rounded based upon the resolution of measurements.

**Attribute**	**Outside**	**Inside**	**Unit**	**Ratio [in/out]**
Temperature	23.1 ± 2.3	27.1 ± 1.3	[°C]	1.17
UVA	19400 ± 10	19390 ± 10	[mW m^{-2}]	1.00
UVB	127 ± 19	31 ± 6	[mW m^{-2}]	0.24
PAR	1270 ± 110	990 ± 140	[µmol m^{-2} s^{-1}]	0.78

Supplementary Table 4. Other possible products detected from the mixture of 10 ml each of 10 mM catechol (benzene-1,2-diol), 20 mM KCl, 50 mM $CuSO_4$ and 50 mM H_2O_2 after 2 hours of reaction. The soluble phase was first extracted by CH_2Cl_2. Measurements conducted by the University of California, Berkeley, QB3/Chemistry Mass Spectrometry Facility[c].

**Method**	**products**	**phase**
laser absorption	CO_2	gaseous
GC/MS	Chloroform ($CHCl_3$)	gaseous
magnet EI^+	furan	soluble
magnet EI^+	tetrahydrofuran	soluble
magnet EI^+	dibutyl phthalate	soluble
magnet EI^+	2-ethyl-1-hexanol	soluble
magnet EI^+	2,4-bis(1,1-dimethylethyl)-phenol	soluble
magnet EI^+	1-(2-hydroxy-6-methoxyphenyl)-ethanone	soluble
magnet EI^+	4'-hydroxy-2,2-dimethylpropiophenone	soluble
magnet EI^+	2,2'-methylenebis[6-(1,1-dimethylethyl)4-ethyl-phenol	soluble
magnet EI^+	7,9-dit-ert-butyl-1-oxaspiro (4,5) deca-6,9-diene-2,8-dione	soluble
TOF MS ES^-	$CuKS_2$	precipitate
TOF MS ES^-	Cu_2S_2	precipitate
TOF MS ES^-	$CuKS_2O_8$	precipitate
TOF MS ES^-	$Cu_2S_2O_8$	precipitate
TOF MS ES^-	$C_7H_6Cu_3K_2S_3$	precipitate
TOF MS ES^-	$C_6H_2O_5Cu_3K_2S_1$	precipitate

[c] About QB3/Chemistry Mass Spectrometry Facility https://qb3.berkeley.edu/facility/msf/

Supplementary Note 1. A simplified calculation on CH_3Br and CH_3Cl emissions with respect to the global agricultural usage of copper(II) sulfate.

The amounts of copper(II) sulfate applied in global agriculture each year: According to Global Copper Sulphate Market Report and Forecast 2021-2026[d], 391.9 million tons of copper(II) sulfate ($CuSO_4$) were produced in 2020, half of which was used as fungicide, herbicide, or pesticide in worldwide agriculture (equivalent to ~1.23×10^{12} mol of $CuSO_4$). Global production of $CuSO_4$ is projected to increase to 498.7 million tons by 2026.

The relationship between copper (II) amendments and methyl halide production: Based upon time-series sunlit experiments (Figure 3 in the main text), 5×10^{-4} mol (10ml, 50mM) of Cu(II) addition to 50 grams of live soil samples yielded 1656 ± 752 ng CH_3Br and 1018 ± 273 ng CH_3Cl, respectively, during the 17 days of ambient light exposure (integral of the flux-time curve), in comparison to the control samples (a.k.a., subtracting background fluxes from control studies).

The following assumptions/approximations are made, and/or caveats exist in this extrapolation: (1) soil samples used in this study are assumed to be representative of global agricultural soils in terms of organic matter (content, composition and structure), chloride and bromide contents and other geochemical properties which may influence the reaction to produce methyl halides; (2) the catalytic efficiency of Cu(II) on methyl halide production lasts ~17 days, as observed in the light experiments, despite that it may last longer without the sunlight attenuation by glass jars; (3) we omitted the error in individual flux measurements for sake of clarity, as these errors are typically much smaller than the standard deviation associated with replicate incubations.

Extrapolation:

CH_3Br = 1656 ± 752 [ng CH_3Br] $\div$ 0.0005 [mol Cu amended] $\times 1.23 \times 10^{12}$ [mol Cu pesticides yr^{-1}] $\div 10^{18}$ [ng Gg^{-1}] = 4.1 ± 1.9 [Gg CH_3Br yr^{-1}];

CH_3Cl = 1018 ± 273 [ng CH_3Cl] $\div$ 0.0005 [mol Cu amended] $\times 1.23 \times 10^{12}$ [mol Cu pesticides yr^{-1}] $\div 10^{18}$ [ng Gg^{-1}] = 2.5 ± 0.7 [Gg CH_3Cl yr^{-1}].

[d] Report retrieved from https://www.expertmarketresearch.com/reports/copper-sulphate-market

Supplementary References

1. Comba, P., Kerscher, M., Krause, T. & Schöler, H. F. Iron-catalysed oxidation and halogenation of organic matter in nature. *Environ. Chem.* **12**, 381–395 (2015).
2. Pracht, J., Boenigk, J., Isenbeck-Schröter, M., Keppler, F. & Schöler, H. F. Abiotic Fe(III) induced mineralization of phenolic substances. *Chemosphere* **44**, 613–619 (2001).
3. Huber, S. G., Kilian, G. & Schöler, H. F. Carbon suboxide, a highly reactive intermediate from the abiotic degradation of aromatic compounds in soil. *Environ. Sci. Technol.* **41**, 7802–7806 (2007).
4. Huber, S. G., Kotte, K., Schöler, H. F. & Williams, J. Natural abiotic formation of trihalomethanes in soil: results from laboratory studies and field samples. *Environ. Sci. Technol.* **43**, 4934–4939 (2009).
5. Keppler, F., Borchers, R., Pracht, J., Rheinberger, S. & Schöler, H. F. Natural formation of vinyl chloride in the terrestrial environment. *Environ. Sci. Technol.* **36**, 2479–2483 (2002).
6. Keppler, F. *et al.* De novo formation of chloroethyne in soil. *Environ. Sci. Technol.* **40**, 130–134 (2006).
7. Keppler, F. *et al.* Formation of volatile iodinated alkanes in soil: results from laboratory studies. *Chemosphere* **52**, 477–483 (2003).
8. Fahimi, I. J., Keppler, F. & Schöler, H. F. Formation of chloroacetic acids from soil, humic acid and phenolic moieties. *Chemosphere* **52**, 513–520 (2003).
9. Althoff, F. *et al.* Abiotic methanogenesis from organosulphur compounds under ambient conditions. *Nat. Commun.* **5**, 4205 (2014).
10. Krause, T., Tubbesing, C., Benzing, K. & Schöler, H. F. Model reactions and natural occurrence of furans from hypersaline environments. *Biogeosciences* **11**, 2871–2882 (2014).
11. Huber, S. G., Wunderlich, S., Schöler, H. F. & Williams, J. Natural abiotic formation of furans in soil. *Environ. Sci. Technol.* **44**, 5799–5804 (2010).

12. M’hemdi, A., Dbira, B., Abdelhedi, R., Brillas, E. & Ammar, S. Mineralization of catechol by Fenton and photo-Fenton processes. *CLEAN – Soil Air Water* **40**, 878–885 (2012).

13. Studenroth, S., Huber, S. G., Kotte, K. & Schöler, H. F. Natural abiotic formation of oxalic acid in soils: results from aromatic model compounds and soil samples. *Environ. Sci. Technol.* **47**, 1323–1329 (2013).

14. Zazo, J. A., Casas, J. A., Mohedano, A. F., Gilarranz, M. A. & Rodríguez, J. J. Chemical pathway and kinetics of phenol oxidation by Fenton’s reagent. *Environ. Sci. Technol.* **39**, 9295–9302 (2005).

15. Jiao, Y. *et al.* Global methyl halide emissions from rapeseed (*Brassica napus*) using life cycle measurements. *Geophys. Res. Lett.* **47**, e2020GL089373 (2020).
